# Supplementary material for: Local Variation of Hashtag Spike Trains and Popularity in Twitter
Source: PLoS One. 2015 Jul 10;10(7):e0131704. doi: 10.1371/journal.pone.0131704 (PMC4498919; doi:10.1371/journal.pone.0131704)
Supplement: S1 File — (ZIP) [file pone.0131704.s001.zip › S1 File Supporting Information/S1_File_PONE-D-15-11694.rtf]

S1 File. Source and format of the supporting data files.Sampled public tweets from Twitter streaming API (https://dev.twitter.com/streaming/overview).Date range: April 30, 2012 to May 10, 2012Data filtering: Users located in FranceAnalyzed data type: Time stamps of all hashtagsData size: About 10 millions tweets, 3 millions tweets including at least one hashtag, and 3 hundred thousands unique hashtags.- TimeStampsHashtags file corresponds to the full list of the hashtag activity and is ranked from the top most used (popular) hashtag to the least ones:     TimeStampsHashtags_PONE-D-15-11694.dat:           Format: hashtag popularity timestamp      popularity = number of times that hashtag appears- RankHashtags file corresponds to full list of the analyzed hashtags ranked from the top most used (popular) hashtag to the least ones:     RankHashtags_PONE-D-15-11694.dat:           Format: hashtag popularity ---Ceyda Sanli, cedaysan@gmail.comRenaud Lambiotte, renaud.lambiotte@unamur.beJune 17, 2015
